# Facial Expression Recognition Based on LDA Feature Space Optimization

**DOI:** 10.1155/2022/9521329

**Published:** 2022-08-29

**Authors:** Fanchen Zheng

**Affiliations:** College of Science, China Agricultural University, Beijing 100083, China

## Abstract

With the development of artificial intelligence, facial expression recognition has become an important part of the current research due to its wide application potential. However, the qualities of the face features will directly affect the accuracy of the model. Based on the KDEF face public dataset, the author conducts a comprehensive analysis of the effect of linear discriminant analysis (LDA) dimensionality reduction on facial expression recognition. First, the features of face images are extracted respectively by manual method and deep learning method, which constitute 35-dimensional artificial features, 128-dimensional deep features, and the hybrid features. Second, LDA is used to reduce the dimensionality of the three feature sets. Then, machine learning models, such as Naive Bayes and decision tree, are used to analyze the results of facial expression recognition before and after LDA feature dimensionality reduction. Finally, the effects of several classical feature reduction methods on the effectiveness of facial expression recognition are evaluated. The results show that after the LDA feature dimensionality reduction being used, the facial expression recognition based on these three feature sets is improved to a certain extent, which indicates the good effect of LDA in reducing feature redundancy.

## 1. Introduction

With the development of the information society, as one of the subjects for human information exchange, the accurate recognition of facial expression will help to improve the efficiency of people's information exchange and to save time and cost. As one of the most primitive ways of information interaction, facial expression has been preserved to this day and plays an irreplaceable role. People's facial expressions can reveal their attitudes to the received information, the emotions toward people or things, and even their inner thoughts. Therefore, the technology of facial expression recognition can be applied to various scenarios, including human-computer interaction, as well as the security and medical fields, such as public security control, fraud behavior analysis, and patient emotion analysis. The method of facial expression recognition has become a research hotspot in the field of computer vision.

Facial expression recognition can be roughly described as analyzing the data of face images by computer, extracting the useful feature information for recognition, and using this information to determine the category of the images. However, as the facial features are extracted and constructed by a variety of feature extraction methods, they are often of high dimensions. If the high-dimensional features are directly used in the subsequent recognition tasks, then it will bring great difficulties for the recognition task. The reason is that although the high-dimensional features contain the complete face information, they have a large number of redundant features. As the number of samples increases, the redundant information will greatly increase the computational resource overhead but reduce the performance of the recognition model. When the amount of data cannot support the feature dimension, a “dimension disaster” will occur. The performance of the recognition model drops greatly or even becomes ineffective. Therefore, it is a key step to reduce the dimension of high-dimensional features in face recognition technology, which has extremely important practical significance.

The accurate recognition of facial expression recognition is inseparable from the extraction of multidimensional features, so redundant features are inevitable. Although the LDA feature dimensionality reduction method has been widely used in the field of face recognition, the previous work had not analyzed the dimensionality reduction effect of different types of feature sets on expressions. Based on the KDEF face public dataset, this study constructs three types of feature sets such as face traditional artificial feature sets, deep learning feature sets, and hybrid feature sets. Furthermore, it performs LDA feature dimension reduction respectively and uses machine learning models to classify the facial expressions. The experimental results show that dimensionality reduction of hybrid features can achieve the best accuracy of facial expression classification, which shows the importance of multidimensional hybrid feature extraction for machine learning tasks.

In this study, it introduces the positioning of human facial key points, the definition of action units, and the construction of the coding system in Section 2. In Section 3, the process of image correction, feature extraction, feature set construction, and feature dimensionality reduction are introduced in detail. In Section 4, the classification effects of different feature sets under different classification methods and dimensionality reduction methods are compared and analyzed. The conclusions and future studies are presented in Section 5.

## 2. Related Work

The key of facial expression recognition is to construct the concise and accurate facial feature descriptors. The method based on the complete face recognition generally uses the traditional feature descriptors, such as Gabor [1], local binary pattern (LBP) [2], and histogram of oriented gradient (HOG) [3], to extract and construct the global feature set of face images. However, in the actual scenes, the face information in the collected images is often incomplete due to various factors, such as the information loss caused by the shooting angle and the face occlusion caused by glasses, masks, or beards. As the method cannot extract enough face information for recognition, it is difficult to improve the recognition accuracy. In this regard, researchers propose a method to extract high-dimensional face features by the key points of the face [4]. The core of this method is to accurately locate the key points of the face and correct them. The research on the facial key points can be traced back to the facial action coding system (FACS) [5]. FACS is developed by Ekman and Friesen to study the correspondence between facial muscle movements and different expressions. It is a system for classifying human facial movements through facial appearance. The movement of the facial muscles is encoded by FACS in accordance with the subtle changes of the face. It is deconstructed into specific action units (AUs) that generate expressions, which are independent but interconnected. The codes of 46 main action units and their definitions are listed in Table 1.

The features of these facial AUs and the main regions they control can not only be used to distinguish faces but also to reflect the related facial expressions [6]. Since then, more studies have begun to optimize the key feature descriptors of faces. Belhumeur et al. [7] used the global model to generate local position information as latent variables, derived a Bayesian objective function to optimize these local positions, and used the literature research method [8] to align the faces. Literature [9] introduced a method for constructing facial feature descriptors based on orthogonal difference-local binary pattern (OD-LBP). First, 3 gray level differences are calculated for each orthogonal location by subtracting the 2 nearest neighbors and the center pixel from their respective orthogonal values. Second, the respective differences of gray levels are counted. Finally, a vector is generated by concatenating the binary patterns produced by the two orthogonal groups. Kazemi and Sullivan [10] proposed a general gradient-boosted framework to learn the set of regression trees. This framework used the set of regression trees to estimate the location of face descriptors directly from a sparse subset of pixel intensities and then extracted the face features according to the location.

In the process of facial expression recognition, in order to extract more comprehensive face features, the feature dimension is often too high because of the high complexity of facial image data. Therefore, it is usually necessary to reduce the dimension of the extracted features first to reduce the feature redundancy. Principal component analysis (PCA) [11] and linear discriminant analysis (LDA) [12] are widely used because of their simplicity and convenience. Principal component analysis (PCA) is a method based on the Karhunen-Loeve (K-L) transform of statistical theory [13]. Mondal and Bag [14] used PCA to reduce the dimension of the extracted features and combined with the minimum distance classification system to realize face recognition. Zhuang and Guan [15] combined Gabor wavelet and PCA for feature extraction, so as to deal with the full illumination of face images. Linear discriminant analysis (LDA) obtains the optimal projection direction by finding the extreme value of the Fisher criterion function, so that after the sample is projected in this direction, the interclass dispersion is the largest and the intraclass dispersion is the smallest. Fisher's criterion function was first proposed by Fisher in 1936. It was originally used to solve the problem of binary classification and then extended to multiclassification. Bodini et al. [16] combined the effectiveness of deep convolution neural network (DCNN) feature with the feature dimensionality reduction capability of LDA to solve the problem of face recognition for a single person and single sample. Benouareth [17] proposed a facial feature extraction method that combines likelihood sufficient dimension reduction (LSDR) and LDA to handle the face recognition under pose and illumination changes.

**Figure 1:**
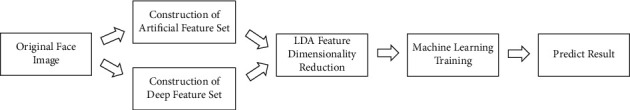
Workflow of facial expression recognition.

Although the LDA feature dimensionality reduction method has been widely used in the field of face recognition, the impact of LDA feature dimensionality reduction on the face recognition under different feature sets has not been evaluated. In this regard, according to the KDEF face public data set, this study conducts a comprehensive analysis for the effect of facial expression recognition based on LDA feature dimensionality reduction, including traditional artificial features, deep learning features, and the hybrid features. First, multidimensional feature extraction is performed. For traditional artificial features, we extract face AU information according to the facial action coding system and construct a 35-dimensional AU feature set. For deep learning features, we use the facial feature extractor based on FaceNet pretraining to extract the deep facial features and construct a 128-dimensional feature set. Second, LDA dimension reduction is performed on the extracted features respectively. Then, machine learning models, such as Naive Bayes and decision trees, are used to analyze the effects of facial expression recognition before and after LDA feature dimensionality reduction. Finally, we compare the effects of PCA and LDA feature dimensionality reduction methods on facial expression recognition. The specific workflow is shown in Figure 1.

## 3. Methods

### 3.1. Face Multidimensional Feature Extraction

In order to fully express the face features, we use different feature extraction methods to extract two aspects of face features according to the KDEF dataset: one is the AU feature based on the artificial methods and the other is the deep feature based on the deep learning.

#### 3.1.1. Face AU Feature Extraction

We adopt the method of AU feature extraction in OpenFace [18] to extract the AU features in face images. As listed in Table 1, the subsets of AU that OpenFace can recognize are as follows: AU01, AU02, AU04, AU05, AU06, AU07, AU09, AU10, AU12, AU14, AU15, AU17, AU20, AU23, AU25, AU26, AU28, and AU45.

**Figure 2:**
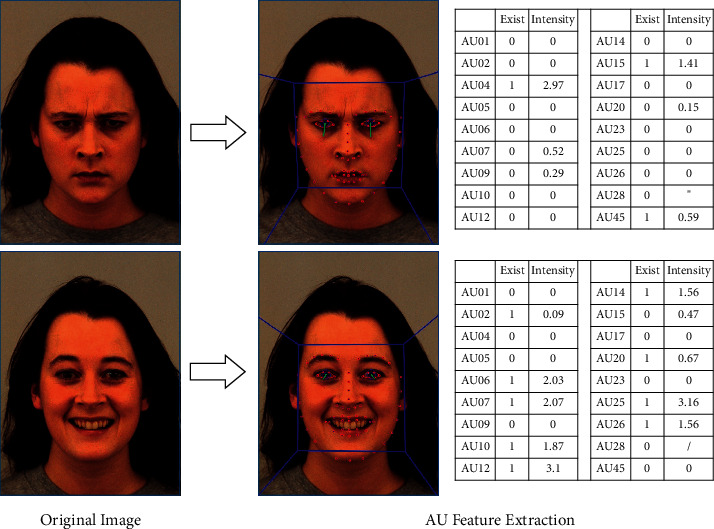
AU feature extraction of face.

OpenFace describes the recognizable AUs in two scoring methods: (1) existence: if the AU is visible on the face, then 0 is absent and 1 is present; (2) intensity: if the AU has the intensity score on the interval [0, 5], then 0 is absent and 5 is present at the maximum intensity. OpenFace is able to judge the existence of 18 recognizable AU subsets and score the intensity of 17 AUs except for AU28. Therefore, each face image can form 35-dimensional AU features, as shown in Figure 2.

#### 3.1.2. Face Deep Feature Extraction

In order to enable the deep learning model to focus on the face and better characterize the face, we use the method described in literature [10] to extract the key points from the dataset and correct the face according to the key points. It uses the gradient boosting decision tree (GBDT) regression method to accurately identify the key points of face. One GBDT is composed of multiple trees. Through these trees, the initial face key points are gradually returned to the real face key points. The specific implementation steps are as follows:

1. There are generally two ways to select the initial face key points. The first one is to take the mean value of real key points of all images as the initial face key points. The second one is to randomly select the real key points of other images as the initial key points of the current image. This study chooses the latter method to select the initial face key points.
2. When a tree is constructed and multiple images are input into the current tree, each image will fall into a leaf node. Calculate the differences between the current face key points and the real face key points of each image, and take the mean value of the differences of all images in the same leaf node as the residual saved by the leaf node.
3. Update the current face key points of each image as the sum of the current face key points and the residual.
4. Repeat steps 2 and 3 until enough trees are established. In that way, the last current face key points can be used as the real face key points.

**Figure 3:**
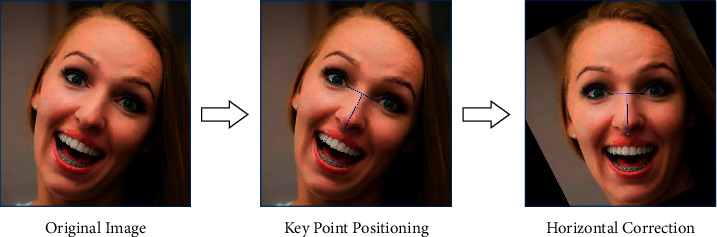
Process of face correction.

According to the above algorithm, we extract 68 face key points for each face image and use these key points to perform the affine transformation correction on the face, so that the corners of the eyes and the nose are close to the horizontal position. It is shown in Figure 3.

**Figure 4:**
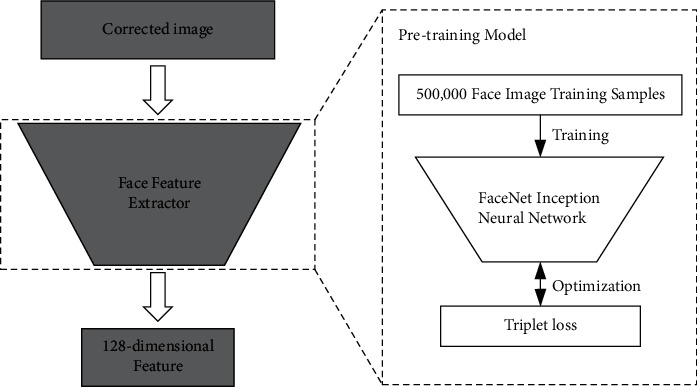
128-dimensional deep feature extraction of face.

After correcting the face image, we use the face feature extractor in OpenFace to extract deep face features. This feature extractor is based on the pretraining deep learning model FaceNet [19], which is shown in Figure 4.

OpenFaces uses 500,000 images to train FaceNet, including the two largest labeled face recognition datasets, CASIA-WebFaces [20], and FaceScrb [21]. It can extract the 128-dimensional deep feature of efficient facial feature expression.

### 3.2. Face Feature Dimensionality Reduction

Due to the diversity of facial expressions, recognizing facial expressions is a multiclassification task, so it needs to use the multiclassification promotion form of LDA. For the problem of multiclassification, we assume the dataset *c*, in which any sample *x*_*i*_ is an n-dimensional vector, and *y*_*i*_ is its category, *y*_*i*_ ∈ {*n*_1_, *n*_2_, ..., *n*_*k*_} , with a total of *k* categories. *N*_*j*_(*j*=1,2,3, ..., *k*) is defined as the number of Class *j* sample, *X*_*j*_(*j*=1,2,3, ..., *k*) is defined as the set of Class *j* sample, *μ*_*j*_(*j*=1,2,3, ..., *k*) is defined as the mean vector of Class *j* sample, and ∑_*j*_(*j*=1,2,3, ..., *k*) is defined as the generalized covariance matrix of Class *j* sample.

Assuming that the matrix formed by the projected hyper-plane basis is *W*=(*w*_1_, *w*_2_, ..., *w*_*d*_), then the optimization objective is as follows:

(1) $$\begin{array}{c} \arg\underset{w}{\max}J\left(W\right)=\frac{w^{T}S_{b}w}{w^{T}S_{w}w}, \end{array}$$

where *S*_*w*_ is the intraclass scatter matrix:

(2) $$\begin{array}{c} S_{w}=\sum_{j=1}^{k}\sum_{j}=\sum_{j=1}^{k}S_{wj}=\sum_{j=1}^{k}\sum_{x\in X_{j}}\left(x-\mu_{j}\right){\left(x-\mu_{j}\right)}^{T}. \end{array}$$

*S*_*b*_ is the interclass scatter matrix:

(3) $$\begin{array}{c} S_{b}=\sum_{j=1}^{k}N_{j}\left(\mu_{j}-\mu \right){\left(\mu_{j}-\mu \right)}^{T}. \end{array}$$

In equation (3),

(4) $$\begin{array}{c} \mu =\frac{1}{m}\sum_{i=1}^{m}x_{i}. \end{array}$$

Since *J*(*W*) is a matrix, the diagonal element product is used to replace the matrix for optimization:

(5) $$\begin{array}{c} \hat{J}\left(W\right)=\frac{\prod_{i=1}^{d}w_{i}^{T}S_{b}w_{i}}{\prod_{i=1}^{d}w_{i}^{T}S_{w}w_{i}}=\prod_{i=1}^{d}\frac{w_{i}^{T}S_{b}w_{i}}{w_{i}^{T}S_{w}w_{i}}. \end{array}$$

In equation (5), the right side of $\hat{J}\left(W\right)$ is the continuous multiplication form of the generalized Rayleigh quotient. It only needs to obtain the maximum value one by one and then *J*(*W*) will obtain the maximum value. As the maximum value of the Rayleigh quotient *w*^*T*^*S*_*b*_*w*/*w*^*T*^*S*_*w*_*w* is the maximum eigenvalue of *S*_*w*_^−1^*S*_*b*_, the matrix W formed by the eigenvectors corresponding to the product of the largest *d* eigenvalues is the mapping result after feature dimensionality reduction.

### 3.3. Introduction of Machine Learning Classification Models

In order to evaluate the influence of the feature set on the face recognition after LDA dimensionality reduction, we rely on the several machine learning methods for facial expression recognition, including Naive Bayes classification model (NB), the decision tree classification model (DT), K-nearest neighbor (KNN), random forest (RF), and support vector machine (SVM). Among them, the Naive Bayes model [22] outputs the probability that a sample belongs to a specific category by predicting the membership probability of the samples and each category. It is assumed that *S*=(*s*_1_, *s*_2_, ..., *s*_*n*_) is an item to be classified and each *s*_*i*_ is a feature attribute of **S**. According to the existing category set *C*=(*c*_1_, *c*_2_, ..., *c*_*m*_), we can calculate the probability of all categories under the characteristic condition of **S** and select the category with the highest probability as the category label of **S**. In this way, we can obtain the equation definition of Naive Bayes classifier as shown in the following equation:

(6) $$\begin{array}{c} V\left(S\right)=\underset{c_{i}\in C}{\arg\max}\frac{p\left(c_{i}\right)\prod_{k=1}^{n}p\left(s_{k}|c_{i}\right)}{p\left(S\right)}. \end{array}$$

In equation (6), *V*(*S*) is the category label of **S**, *p*(*S*) is a constant for all categories, and *p*(*c*_*i*_) is the category prior probability. *p*(*s*_1_*|c*_*i*_), *p*(*s*_2_*|c*_*i*_), ..., *p*(*s*_*n*_*|c*_*i*_) is the conditional probability of each feature attribute in **S** under the condition of category *c*_*i*_. All of these can be obtained from the training sample sets.

The decision tree classification model [23] consists of three parts: root node, internal node, and leaf node. The root node is the starting point of decision-making, and a decision path can be formed from the root node to a leaf node. Each of the internal nodes contains a feature that can divide the data. Each leaf node corresponds to a category. We use the classic algorithm ID3 to build a decision tree model. We suppose that *S* is the set of training samples, |*S*| is the number of training samples, and *A* is any feature of the sample. When the samples are divided into *n* different classes *C*_1_, *C*_2_,…, *C*_*n*_, and the number of samples in these classes is marked as |*C*_1_|, |*C*_2_|,…, |*C*_*n*_|, then the probability that any sample S belongs to class *C*_*i*_ is as follows:

(7) $$\begin{array}{c} p\left(S_{i}\right)=\frac{\left|C_{i}\right|}{\left|S\right|}. \end{array}$$

Then, the total information entropy is as follows:

(8) $$\begin{array}{c} \text{Entropy}\left(S\right)=-\sum_{\left|S\right|}\sum_{n}p\left(S_{i}\right)\log p\left(S_{i}\right). \end{array}$$

The information entropy of samples divided in accordance with Feature *A* is as follows:

(9) $$\begin{array}{c} \text{Entropy}\left(S,A\right)=\sum_{A}\left(\frac{\left|S_{v}\right|}{\left|S\right|}\right)\text{Entropy}\left(S_{v}\right). \end{array}$$

The information gain of Feature *A* on the set *S* is as follows:

(10) $$\begin{array}{c} \text{Gain}\left(S,A\right)=\text{Entropy}\left(S\right)-\text{Entropy}\left(S,A\right). \end{array}$$

In equation (9), ∑_*A*_ is the sum of all possible values *v* of attribute *A*, *S*_*v*_ is the subset of sample features *A* with *v* value in *S*, and |*S*_*v*_| is the number of samples in *S*_*v*_. According to the theory of information theory, the ID3 algorithm uses “information gain” to measure uncertainty; that is, the greater the information gain, the smaller the uncertainty and the better the division effect.

## 4. Experimental Results and Analysis

The experimental conditions in this study are CPU: Intel(R) Core (TM) i5-10210U, RAM: 16.0 GB, and the programming language is Python 3.6.

### 4.1. Datasets and Evaluation Metrics

**Figure 5:**
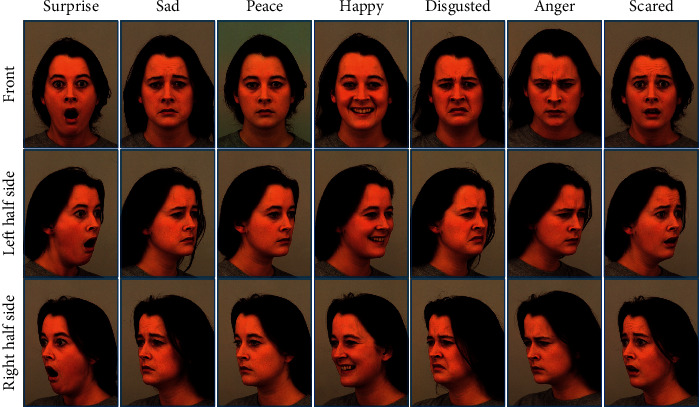
Samples in the KDEF.

The experiments in this study are based on the KDEF public face dataset [24]. The KDEF dataset was originally used for psychological and medical experiments on perception, attention, emotion, memory, etc. It contains the facial information of 70 people, 35 males and 35 females, who are between the ages of 20 and 30. They have no beards, earrings, or glasses and have removed their makeup as far as possible before shooting. In the process of collecting the dataset, all the subjects wore special gray T-shirts, and the light was soft indirect light, evenly distributed on both sides of the face. Seven different expressions are collected for each person, including surprise, sadness, happiness, etc., and each expression has the color images from 5 angles. The entire dataset has a total of 4900 images, each with a size of 562 ∗ 762 pixels. Among them, there are 2916 photos that can identify the frontal and half-side face. Some samples are shown in Figure 5.

We randomly select 10% of the identifiable samples as the test set and the rest as the training set. The recognition effect is measured by the multiclassification evaluation metrics, including microprecision (micro-P), microrecall (micro-R), micro-F1, macroprecision (macro-P), macrorecall (macro-R), and macro-F1, which is shown in equations (11)–(16), where *N* denotes the number of categories, TP is true positive, FP is false positive, and FN is false negative.

(11) $$\begin{array}{c} \text{micro}‐\text{Precision}=\frac{\sum_{i=1}^{N}TP_{i}}{\sum_{i=1}^{N}TP_{i}+\sum_{i=1}^{N}FP_{i}}, \end{array}$$

(12) $$\begin{array}{c} \text{micro}‐\text{Recall}=\frac{\sum_{i=1}^{N}TP_{i}}{\sum_{i=1}^{N}TP_{i}+\sum_{i=1}^{N}FN_{i}}, \end{array}$$

(13) $$\begin{array}{c} \text{micro}‐F1=2\frac{\text{micro}-Recall\times micro-\text{Precision}}{\text{micro}-\text{Recall}+\text{micro}-\text{Precision}}\text{,} \end{array}$$

(14) $$\begin{array}{c} \text{macro}‐\text{Precision}=\frac{1}{N}\sum_{i=1}^{N}\frac{TP_{i}}{TP_{i}+FP_{i}}, \end{array}$$

(15) $$\begin{array}{c} \text{macro}‐\text{Recall}=\frac{1}{N}\sum_{i=1}^{N}\frac{TP_{i}}{TP_{i}+FN_{i}}, \end{array}$$

(16) $$\begin{array}{c} \text{macro}‐F1=\frac{1}{N}\sum_{i=1}^{N}F1_{i}. \end{array}$$

### 4.2. Analysis of LDA Dimensionality Reduction Effect for Facial Expression Recognition

In order to test the effect of LDA feature dimensionality reduction on the accuracy of facial expression recognition, we respectively extract the AU feature and the 128-dimensional feature from the images in the dataset as two different feature sets and regard the hybrid feature set as the third feature set. For the three feature sets, LDA was used to reduce the dimensions to 7 dimensions, and the training set of the machine learning method is used to test its classification effect. Therefore, the experiment can be divided into three parts: analysis of LDA dimensionality reduction effect based on AU features, analysis of LDA dimensionality reduction effect based on 128-dimensional face deep features, and analysis of LDA dimensionality reduction effect based on hybrid features.

#### 4.2.1. Analysis of LDA Dimensionality Reduction Effect Based on AU Features

The facial AU feature is obtained by dividing the face into regions and counting the influence of each expression on different regions. It contains rich expression information, but it may also have redundancy that affects the recognition results. Therefore, we adopt several machine learning methods for facial expression recognition on basis of the AU feature and compare the recognition effects before and after LDA feature dimensionality reduction. The results are listed in Table 2.

Macro-F1 is taken as an example in Table 2. After the LDA dimensionality reduction, the macro-F1 of the NB and DT increased, while that of KNN, RF, and SVM decreased. This shows that there is some redundancy in the original AU feature set.

#### 4.2.2. Analysis of LDA Dimensionality Reduction Effect Based on 128-Dimensional Deep Features

The feature extraction based on the deep learning model can perform a higher-level abstract expression. In contrast to the local AU features, the semantic information of these features is richer, but the detailed location information is relatively brief. The facial expression recognition effect of 128-dimensional deep features and the dimensionality reduction effect of LDA are listed in Table 3.

It can be seen from Table 3 that the macro-F1 of facial expression recognition based on the original face 128-dimensional deep features is low. After LDA dimensionality reduction, the macro-f1 of all the methods is significantly improved except for the RF method.

The reason is that most of the original 128-dimensional deep features are facial semantic information, which has little effect on the facial expression recognition that requires detailed information. Therefore, the recognition effect before dimensionality reduction is low. LDA can project the feature to the largest distance between classes according to the label, which can also be regarded as mapping the 128-dimensional deep features to the feature space that can conduct the facial expression recognition. Due to the high expressive ability of deep features, these features can better express facial expression information after dimensionality reduction. Therefore, compared with face AU features, the 128-dimensional deep features can achieve higher recognition accuracy after dimensionality reduction.

#### 4.2.3. Analysis of LDA Dimensionality Reduction Effect Based on Hybrid Features

We spliced local face AU features and 128-dimensional deep features containing more global semantic information to obtain the hybrid features. The facial expression recognition effect of hybrid features is listed in Table 4.

As listed in Table 4, compared with the above single feature set, the hybrid features can achieve higher recognition accuracy before and after LDA dimensionality reduction. After dimensionality reduction, the macro-F1 of the NB, DT, KNN, RF, and SVM methods increased by 19.22%, 18.04%, 15.10%, 3.91%, and 11.83%, respectively. The reason is that the hybrid features contain multidimensional face features, namely, local face AU features and global 128-dimensional deep semantic features. It enables the model to be fully learned, so as to achieve the higher recognition accuracy.

Combining Tables 2–4, it can be seen that no matter which feature set is used, LDA dimensionality reduction can effectively improve the recognition accuracy of the two machine learning methods. The main reason is that LDA feature dimensionality reduction increases the correlation between features and categories; that is, the class spacing is maximized while the intraclass dispersion is minimized, so that the subsequent model can better learn the interclass differences, resulting in the higher recognition evaluation matrix. The dimensionality reduction efficiencies of LDA for AU features, 128-dimensional deep features, and hybrid features are 15.14 s, 29.85 s, and 224.3 s, respectively.

### 4.3. Comparison of Different Dimensionality Reduction Methods

**Figure 6:**
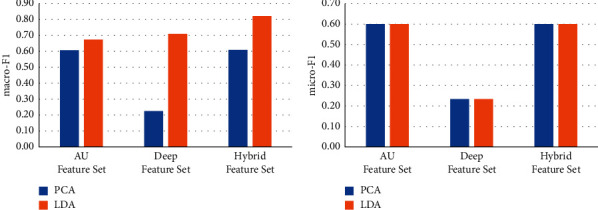
Comparison of the effects of different feature dimensionality reduction methods based on the NB.

**Figure 7:**
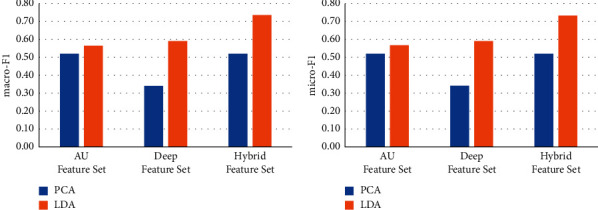
Comparison of the effects of different feature dimensionality reduction methods based on the DT.

**Figure 8:**
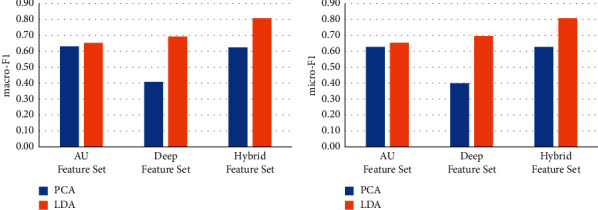
Comparison of the effects of different feature dimensionality reduction methods based on the KNN.

**Figure 9:**
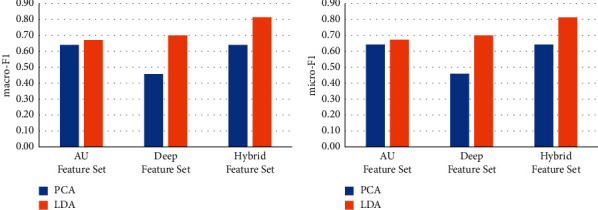
Comparison of the effects of different feature dimensionality reduction methods based on the RF.

**Figure 10:**
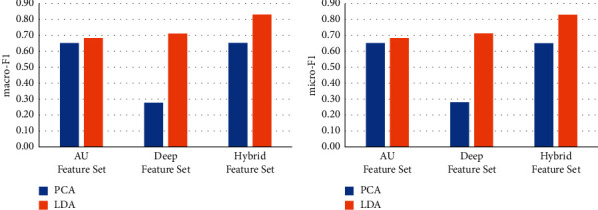
Comparison of the effects of different feature dimensionality reduction methods based on the SVM.

Principle component analysis (PCA) has been widely used as a classic method of unsupervised dimensionality reduction. Its main idea is to use the covariance matrix of the data to calculate the eigenvalues. The eigenvalues are arranged from large to small, and the feature vectors corresponding to several largest representative components (features) are used to represent the overall characteristics of the data, so as to achieve the purpose of feature dimensionality reduction. We use LDA and PCA respectively to reduce the feature dimension for different feature sets and keep the same feature dimension after dimensionality reduction. We use the dimensionality-reduced features for the training of machine learning models to test the effect on face recognition. The results are shown in Figures 6–10.

It can be seen from Figures 6–10 that for different types of feature sets and different machine learning methods, the accuracy of LDA feature dimensionality reduction for face recognition is higher than that of PCA method. Furthermore, the standard deviation of the correct samples identified by LDA is generally lower than that of PCA method, which indicates that LDA feature dimensionality reduction can make the feature be of greater category expression ability. Combining the ACC without dimensionality reduction in Tables 2–4, it can be seen that the accuracy of face recognition after PCA dimensionality reduction is even lower than that before dimensionality reduction. The reason is that PCA is an unsupervised data dimensionality reduction method. As this method does not use category information, it cannot obtain the effective dimensionality reduction effect for complex face feature sets. LDA is a supervised data dimensionality reduction method. It can be label oriented and select the vector that makes the Fisher criterion function reach the extreme value as the best projection direction, so that the sample can achieve the largest interclass dispersion and the smallest intraclass dispersion after being projected in this direction.

## 5. Conclusion and Discussion

The accurate recognition of facial expression recognition is inseparable from the extraction of multidimensional features, but there is often a lot of redundancy in multidimensional features. Based on the KDEF face public dataset, this study evaluates the effect of LDA feature dimensionality reduction method on facial expression recognition. First, the extraction of multitype features is performed on the face, including 35-dimensional face AU features based on artificial construction, 128-dimensional face deep features based on the FaceNet pretraining model, and 163-dimensional hybrid features. Second, LDA was used to reduce the dimensions of three different feature sets to 7 dimensions. Then, the classical machine learning methods are used to evaluate the effect of facial expression recognition before and after feature dimensionality reduction. Finally, we compare the LDA and PCA feature dimensionality reduction methods. The experimental results show that after the LDA feature dimensionality reduction, the facial expression recognition effects of the three feature sets have been improved to a certain extent, which are better than the PCA method. The dimensionality reduction of hybrid features has the best effect on improving the effect of facial expression recognition, with the macro-F1 reaching 82.90%. In addition, we found that no matter whether the feature dimensionality reduction is carried out or what machine learning method is used, the hybrid features can achieve a higher recognition accuracy than a single one. It shows that it is important to extract the multidimensional hybrid features in facial expression recognition.

As this study only compares the same number of dimensions when reducing the dimension, it may need to dynamically adjust the feature dimensions for different feature sets. Hence, in the future, we will explore the effect of the feature dimensionality reduction under the optimal number of dimensions. The research on the effect of feature dimensionality reduction will be further expanded from multiple directions, such as more machine learning methods, more feature dimensionality reduction methods, and more different types of datasets.

## Figures and Tables

**Table 1:** Codes and definitions of main action units.

Action unit	Definition	Action unit	Definition	Action unit	Definition
AU01	Raise the inner eyebrows	AU13	Pull the corners of the mouth up	AU25	Separate the lips to expose the teeth
AU02	Raise the outer eyebrows	AU14	Shrink the corners of the mouth towards the teeth	AU26	Separate the lips to see the tongue
AU04	Droop the eyebrows overall	AU15	Pull the corners of the mouth down vertically	AU27	Separate the lips to see the throat
AU05	Raise the upper eyelids	AU16	Pull the lower lip down	AU28	Suck the lips to cover the teeth
AU06	Lift up the cheeks	AU17	Squeeze the lower lip upward	AU41	Droop the upper eyelid slightly
AU07	Constrict the eyes	AU18	Wrinkle the mouth in the middle	AU42	Droop the upper eyelid
AU09	Shrink and lift the nose	AU20	Pull the lips backwards	AU43	Close the eyes
AU10	Raise the upper lip	AU22	Purse the lips into a funnel	AU44	Lift the lower eyelid up
AU11	Deepen the middle nasolabial fold	AU23	Tighten the lips into a line	AU45	Blink two eyes
AU12	Raise the corners of mouth	AU24	Squeeze the lips together	AU46	Blink one eye

**Table 2:** LDA dimensionality reduction effect of AU feature.

Method	NB		DT		KNN		RF (*n* = 100)		SVM	
LDA	Before	After	Before	After	Before	After	Before	After	Before	After
Micro-P	0.5833	0.6756	0.5568	0.5667	0.6604	0.6517	0.6956	0.6699	0.7038	0.6815
Macro-P	0.5446	0.6731	0.5536	0.5640	0.6535	0.6527	0.6975	0.6724	0.7054	0.6840
Micro-R	0.5468	0.6743	0.5546	0.5650	0.6553	0.6541	0.6991	0.6737	0.7071	0.6854
Macro-R	0.5446	0.6731	0.5536	0.5640	0.6535	0.6527	0.6975	0.6724	0.7054	0.6840
Micro-F1	0.5644	0.6749	0.5557	0.5659	0.6578	0.6529	0.6974	0.6718	0.7054	0.6834
Macro-F1	0.5446	0.6731	0.5536	0.5640	0.6535	0.6527	0.6975	0.6724	0.7054	0.6840

**Table 3:** LDA dimensionality reduction effect of 128-dimensional deep features.

Method	NB		DT		KNN		RF (*n* = 100)		SVM	
LDA	Before	After	Before	After	Before	After	Before	After	Before	After
Micro-P	0.4459	0.7075	0.3722	0.5903	0.6557	0.6965	0.7642	0.6986	0.6336	0.7121
Macro-P	0.4439	0.7067	0.3709	0.5889	0.6131	0.6939	0.7644	0.6983	0.6309	0.7119
Micro-R	0.4442	0.7076	0.3715	0.5894	0.6141	0.6948	0.7664	0.6993	0.6328	0.7127
Macro-R	0.4439	0.7067	0.3709	0.5889	0.6131	0.6939	0.7644	0.6983	0.6309	0.7119
Micro-F1	0.4450	0.7075	0.3719	0.5899	0.6342	0.6957	0.7653	0.6989	0.6332	0.7124
Macro-F1	0.4439	0.7067	0.3709	0.5889	0.6131	0.6939	0.7644	0.6983	0.6309	0.7119

**Table 4:** LDA dimensionality reduction effect of hybrid features.

Method	NB		DT		KNN		RF (*n* = 100)		SVM	
LDA	Before	After	Before	After	Before	After	Before	After	Before	After
Micro-P	0.6725	0.8212	0.5570	0.7374	0.6649	0.8089	0.7740	0.8143	0.7089	0.8296
Macro-P	0.6283	0.8205	0.5545	0.7349	0.6576	0.8086	0.7748	0.8139	0.7107	0.8290
Micro-R	0.6298	0.8213	0.5558	0.7357	0.6595	0.8096	0.7764	0.8147	0.7123	0.8298
Macro-R	0.6283	0.8205	0.5545	0.7349	0.6576	0.8086	0.7748	0.8139	0.7107	0.8290
Micro-F1	0.6505	0.8212	0.5564	0.7341	0.6622	0.8092	0.7752	0.8145	0.7106	0.8297
Macro-F1	0.6283	0.8205	0.5545	0.7349	0.6576	0.8086	0.7748	0.8139	0.7107	0.8290

## Data Availability

The data used to support the findings of this study are available from the corresponding author upon request.
